# Antithrombotic Management during Percutaneous Mitral Valve Repair with the Mitraclip System in a Patient with Heparin-Induced Thrombocytopenia

**DOI:** 10.1055/s-0038-1675586

**Published:** 2018-11-10

**Authors:** Francesco Saia, Elena Biagini, Alessandra Berardini, Matteo Chiarabelli, Emanuela Bertolino, Miriam Compagnone, Claudio Rapezzi

**Affiliations:** 1 Cardiology Unit, Cardio-Thoraco-Vascular Department, University Hospital of Bologna, Policlinico S. Orsola-Malpighi, Bologna, Italy

**Keywords:** Thrombosis, heart, antithrombin

## Abstract

Interventional cardiology procedures require full anticoagulation to prevent thrombus formation on catheters and devices with potential development of embolic complications. Bivalirudin, a short half-life direct thrombin inhibitor, has been largely used during percutaneous coronary interventions and represents the preferred alternative to heparin in patients with heparin-induced thrombocytopenia (HIT). However, few data are available about intraprocedural use of bivalirudin during transcatheter structural heart disease interventions. Activated clotting time (ACT) monitoring during bivalirudin infusion presents some limitations and it is not mandatory. We report a case of bivalirudin use in a patient with type-2 HIT during percutaneous mitral valve repair with the Mitraclip system (Abbott, Abbott Park, Illinois, United States). Despite use of standard bivalirudin dose (0.75 mg/kg bolus and 1.4 mg/kg/min infusion—reduced infusion rate was motivated by a glomerular filtration rate of 37 mL/min), the patient developed a large thrombus on the second clip during its orientation toward the mitral orifice. ACT was measured at that time and was suboptimal (240 seconds). The case was successfully managed with clip and thrombus retrieval, adjunctive 0.3 mg/kg bivalirudin bolus and increased infusion rate, and clip repositioning with ACT monitoring. This report makes the case for mandatory ACT checking and drug titration during high-risk catheter–based structural heart disease interventions, even when thromboprophylaxis is performed with bivalirudin. Additional coagulation tests may be useful to monitor bivalirudin response in similar cases.

## Introduction

Percutaneous mitral valve repair with the Mitraclip system (Abbott, Abbott Park, Illinois, United States) has recently emerged as a safe and effective procedure to treat patients with degenerative mitral regurgitation (MR) with high surgical risk and a technically favorable anatomy. The Mitraclip is being extensively used also to treat functional MR in patients with persisting heart failure symptoms despite optimal medical treatment and, when indicated, cardiac resynchronization therapy (CRT). 1 The guiding catheter is advanced from the femoral vein to the left atrium through a transseptal approach. Then, guided by transesophageal echocardiography (TEE), one or more clips are placed on the mitral valve leaflets through a steerable delivery system, to permanently approximate the leaflets and create a double valve orifice.

Interventional cardiology procedures require full anticoagulation to prevent thrombus formation on catheters and devices with potential development of embolic complications. The Mitraclip procedure implies the long permanence of bulky devices into the left atrium, a chamber characterized by low blood flow velocities, especially in patients with atrial fibrillation and a dilated atrium. Hence, thrombotic risk is high and intraprocedural antithrombotic management must be very accurate. In general, an initial bolus of heparin is given after transseptal puncture and activated clotting time (ACT) is monitored to administer additional doses and maintain an ACT value greater than 250 seconds.

Heparin-induced thrombocytopenia (HIT) is a potentially catastrophic immune-mediated complication of heparin caused by antibodies to complexes of platelet factor-4 (PF4) and heparin. 2 HIT predisposes to thrombosis because platelets release microparticles that activate thrombin. In patients undergoing percutaneous coronary intervention (PCI) with a diagnosis of HIT, intraprocedural administration of bivalirudin is recommended.

We report the challenging management of a patient with HIT who underwent Mitraclip procedure with bivalirudin infusion.

## Case Presentation

**Fig. 1:**
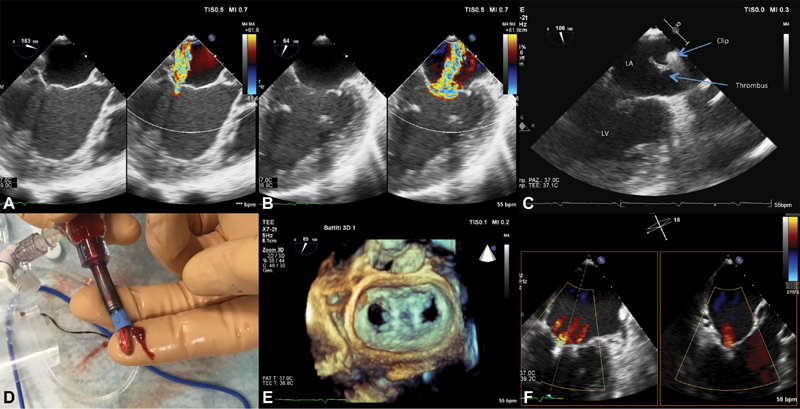
Intraprocedural echocardiography and clip thrombosis. ( **A** ) Intraprocedural transesophageal echocardiography (TEE) color Doppler at the left ventricular outflow tract view showing severe mitral regurgitation (MR). ( **B** ) TEE at the intercommissural view showing the extension of severe MR to the entire mitral valve orifice. ( **C** ) Thrombus adherent to the clip during the maneuvers to orient the second clip toward the mitral orifice. LA, left atrium; LV, left ventricle. ( **D** ) Picture of the clip and the thrombus once retrieved from the patient. ( **E** ) Double orifice of the mitral valve at 3D echocardiography after implantation of the two clips. ( **F** ) Final procedural result with residual trivial mitral regurgitation, X-plane view.

An 81-year-old woman with dilated cardiomyopathy was referred to our institution for urgent Mitraclip procedure because of severe MR and heart failure refractory to full medical treatment, including high-dose intravenous (IV) diuretics. Echocardiography showed severe left ventricular dilatation, with 20% ejection fraction, severe left and right atrial enlargement, and severe functional mitral and tricuspid regurgitation ( Fig. 1 ). Comorbidity included type 2 HIT, chronic hepatitis C virus infection, chronic anemia, chronic kidney disease (CKD) with glomerular filtration rate 37 mL/min, previous mammary cancer followed by radiotherapy (40 years before), and previous operated colon adenocarcinoma (10 years before). Baseline activated partial thromboplastin time (aPTT), international normalized ratio (INR), and fibrinogen were within normal limits. The patient had received CRT-D 4 years earlier. *Ongoing therapy* : furosemide 500 mg/die IV, carvedilol 6.25 mg bid, potassium canrenoate 100 mg, iron supplement, pantoprazole, levothyroxine, and levosimendan.

The Mitraclip procedure was conducted with standard technique. Saline solution without heparin was used for catheters' flushing. We administered bivalirudin bolus (0.75 mg/kg IV) immediately after transseptal puncture and at the same time a PCI-dose infusion of 1.40 mg/kg per hour was started (recommended dose reduction for CKD).

After release of the first clip, during positioning of a second clip to improve procedural result, a moving image suggestive for thrombus became evident on top of the clip ( Fig. 1 and Video 1 ). ACT measured 240 seconds. An additional bivalirudin bolus of 0.3 mg/kg was administered and the infusion rate increased to 1.75 mg/kg/hour. The mass did not reduce and actually tended to increase. Hence, the clip was carefully removed to avoid embolization. Once outside the catheter, a large thrombus attached to the clip was evident ( Fig. 1 , panel D). Then, a line for additional bivalirudin continuous infusion (and flushing) was placed directly on the guiding catheter, at a rate of 0.2 mg/kg/hour. ACT then measured 455 seconds. The procedure was successfully completed with reduction of MR grade from 4+ to 1+ . Bivalirudin infusion was stopped and the guiding catheter removed without complications. No clinical signs of embolization appeared, although we cannot rule out small silent systemic embolizations. The patient was discharged home after 1 week. Antithrombotic regimen was lifelong aspirin and clopidogrel for 6 months.

**Video 1**

Intraprocedural transesophageal echocardiography showing the sessile thrombus attached to the clip. Online content including video sequences viewable at: www.thieme-connect.com/products/ejournals/html/10.1055/s-0038-1675586 .

## Discussion

Bivalirudin is a direct thrombin inhibitor with short half-life which has been widely used for anticoagulation in the setting of PCI, and represents the first-line intraprocedural anticoagulant for patients with HIT. It is administered with a 0.75-mg/kg bolus, followed by infusion of 1.75 mg/kg/hour in patients with GFR greater than 60 mL/min, reduced to 1.40 mg/kg/min in patients with GFR 30 to 59 mL/min. An infusion at 0.25 mg/kg/hour can be prolonged 4 to 12 hours if clinically indicated. ACT can be used to monitor effective bivalirudin administration, but it is not routinely recommended. Safety and efficacy of bivalirudin in structural heart disease procedures have been poorly investigated. The Effect of Bivalirudin on Aortic Valve Intervention Outcomes-3 (BRAVO-3) randomized trial demonstrated noninferiority of bivalirudin compared with heparin in rates of major bleeding or net adverse cardiovascular events during transcatheter aortic valve implantation. 3 Yet, flow in the left ventricular outflow tract is torrential and thrombotic risk is supposedly lower than that in the left atrium. There are a few reports about successful bivalirudin use during ablation procedures in patients with HIT. 4 5 To the best of our knowledge, this is the first report of bivalirudin use in a patient undergoing a Mitraclip procedure.

Despite the use of recommended bivalirudin dose, our patient developed a large thrombus on the clip during the procedure. ACT was measured only at that time and was actually lower than expected. The procedure was successfully completed giving an additional 0.3 mg/kg bolus, as per indications for use, and arbitrary continuous catheter flushing with a low bivalirudin dose.

Limitations of ACT monitoring of bivalirudin therapy have been previously demonstrated, 6 and there is no guidance regarding what value of ACT would warrant an additional bolus.

Accordingly, routine monitoring of ACT during PCI is not mandatory and is not usually performed. However, the instructions for use suggest to check ACT 5 minutes after the bolus and to administer additional 0.3 mg/kg bolus dose “if needed.” In our case, this was necessary to achieve effective thromboprophylaxis. We also increased infusion rate and continuously flushed the guiding catheter with low-dose bivalirudin.

Differently from heparin, bivalirudin binds directly to thrombin without the need of a cofactor and therefore exhibits predictable and dose-dependent anticoagulation. While bivalirudin activity is independent of antithrombin deficiency, increased heparin clearance, and increased heparin binding proteins, which are among the causes of heparin resistance, other resistance mechanisms such as heparin have been hypothesized, including elevated factor VIII and fibrinogen. 7 Furthermore, because bivalirudin inhibits both free and clot-bound thrombin, in the presence of large clot burden higher dosing might be required because of the larger number of binding sites. 7 In our patient, fibrinogen was normal and there was no clinical evidence of vascular thrombosis. Additional coagulation tests were not performed; so, we cannot rule out abnormality of factor VIII level or the presence of unrecognized vascular thrombosis.

Interestingly, bivalirudin resistance (defined as ACT < 300 seconds) was reported in 2.4% of patients undergoing PCI in a single-center study. 8 While bleeding complications were not more common in bivalirudin hyperresponders (ACT > 800 seconds), thrombotic complications were numerically higher in bivalirudin hyporesponders. 8 Overall, the therapeutic window seems wide enough to allow additional bivalirudin doses in suspected bivalirudin hyporesponders, based on ACT values. Remarkably, however, monitoring of direct thrombin inhibitor's efficacy would require more sensitive markers, such as the chromogenic anti-Xa level for heparin, dilute thrombin time, Ecarin thrombin time, and specific chromogenic substrate-based assays, although clinical applicability is limited because of the lack of widely available commercial products. 7

## Conclusion

This case suggests that bivalirudin may be used in place of heparin in patients with HIT undergoing Mitraclip procedure. However, bivalirudin might be subject to resistance mechanisms similar to those previously described in patients receiving heparin, and careful ACT monitoring should be mandatory to verify achievement and permanence in the therapeutic range. When possible, additional coagulation tests should be used. Flushing of guiding catheters and devices with low-dose bivalirudin may represent a useful adjunctive measure. More data are needed to clarify if different ways of monitoring bivalirudin therapy are required for transcatheter structural heart disease interventions. Importantly, this is a single clinical case and our observations should be interpreted cautiously.
